# Biphasic anaphylaxis: a systematic review of the literature

**DOI:** 10.1186/1710-1492-10-S1-A5

**Published:** 2014-03-03

**Authors:** Douglas P Mack

**Affiliations:** 1 Department of Pediatrics, McMaster University, Hamilton, Ontario, Canada

## Background

Biphasic anaphylaxis is a poorly understood allergic phenomenon with significant variation in causative agent, time to onset, outcome and overall frequency. The aim of this review is to better determine the clinical characteristics of this type of allergic reaction.

## Methods

A systematic review was performed identifying case reports and retrospective and prospective studies reporting biphasic allergic reactions. “Biphasic anaphylaxis” (BA) was defined as an anaphylactic reaction consisting of 2 distinct phases separated by at least 1 hour, with both phases meeting internationally recognized diagnostic criteria for anaphylaxis. Pediatric and adult cases were evaluated. Biphasic and uniphasic data was compared using the Chi^2^ test.

## Results

28 articles included descriptions of patients having biphasic reactions with sufficient information for evaluation of clinical characteristics. In total, 150 patients were identified as having biphasic reactions. 84 of these patients met clinical criteria for true BA. Of these, 28 were pediatric cases and 56 were adult. Overall frequency of biphasic reactions was 7.36% of anaphylactic reactions with prospective studies reporting a frequency of 9.07%. Mean time to the second phase of BA was 8.13 hours (95% CI, 6.13-10.14) with similar times between both pediatric and adult populations. The range of reported times to onset of the second phase of biphasic reactions was 1 – 72 hours. 8 articles allowed comparison of medication use and likelihood of biphasic reactions suggesting that the use of epinephrine in the initial treatment may predict the presence of the biphasic reaction (p=0.19). Fatal biphasic reactions were described in 5 patients. Significant limitations were encountered because of inconsistent definition of BA and inadequate reporting of individual patient data.

## Conclusions

Biphasic reactions are common presentations of anaphylaxis with both pediatric and adult patients being affected. Mean and median times to onset of the second phase are variable, but are longer than most emergency department observation time recommendations. Carefully designed prospective studies with clear definitions of BA are necessary to accurately determine the characteristics of these life-threatening reactions.

